# Electrodeposited Organic Layers Formed from Aryl Diazonium Salts for Inhibition of Copper Corrosion

**DOI:** 10.3390/ma10030235

**Published:** 2017-02-28

**Authors:** Ana Chira, Bogdan Bucur, Gabriel-Lucian Radu

**Affiliations:** National Institute of Research and Development for Biological Sciences, Centre of Bioanalysis, 296 Splaiul Independentei, 060031 Bucharest, Romania; ana_chira@yahoo.com (A.C.); glradu2006@gmail.com (G.-L.R)

**Keywords:** copper, diazonium salts, quartz crystal microbalance, Tafel polarization, electrochemical noise

## Abstract

Copper substrates deposed on a gold screen-printed electrode were covered with different aryl diazonium salts by electrodeposition at 0.25 mA for 30 or 300 s. Seven compounds were investigated: 4-aminophenylacetic acid, 4-aminophenethyl alcohol, 4-fluoroaniline, 4-(heptadecafluorooctyl)aniline, 4-aminoantipyrine, 4-(4-aminophenyl)butyric acid and 3,4,5-trimethoxyaniline. Quantitative monitoring of the electrodeposition process was carried out by electrogravimetry using quartz crystal microbalance (QCM). The electrodeposited mass varies between 26 ng/cm^2^ for 4-fluoroaniline formed during 30 s to 442 ng/cm^2^ for 4-phenylbutyric acid formed during 300 s. The corrosion inhibition properties of aryl-modified layers have been studied in buffer citrate with pH = 3 or 3.5% NaCl solutions using electrochemical noise (ECN) and Tafel potentiodynamic polarization measurements. A corrosion inhibiting efficiency up to 90% was found. The highest corrosion inhibition was obtained for 4-(4-aminophenyl)butyric acid and the lowest for 4-fluoroaniline. A relation between the inhibition efficiency and the chemical nature of the substituents in the protective layer was found.

## 1. Introduction

Corrosion is a degradation process of a metal surface resulting from its interaction with the environment or a variety of chemical products. Among the numerous methods used for corrosion prevention, the use of chemical inhibitors remains the most cost effective and practical method [1]. The covering of the metal surface with a protective thin film can act in different ways: blocking of active sites on the surface, reaction with a potential corrosive components, changing media characteristics and/or producing passivizing layers, having as a final effect the reduction of the corrosion rate [2].

Copper is a material commonly used due to its excellent electrical, thermal, or mechanical proprieties although it is susceptible to corrosion [1]. Various inorganic [3] or organic [4] compounds added in working media were proved to be effective inhibitors of the copper corrosion [5]. The formation of organic films on copper surfaces was also tested for corrosion inhibition [6]. The inhibition efficiency is strongly dependent on the structure, chemical properties, concentration or electrode coating process of the organic species formed on the copper surface [7]. The protective layers reported for copper inhibition are formed with different derivatives such as: azoles [8], thiols [9], amines [10], or amino acids [11]. One method that allows the formation of strongly bounded organic layers on copper surface is based on reduction of aryl diazonium salts [12]. The tests were conducted in aerated condition to avoid diffusion controlled reaction. Copper corrosion depends on the nature of the environment, most studies are carried out in oxygen containing media [3,4,5,6,7,8,9,10,11]. 

**Figure 1:**
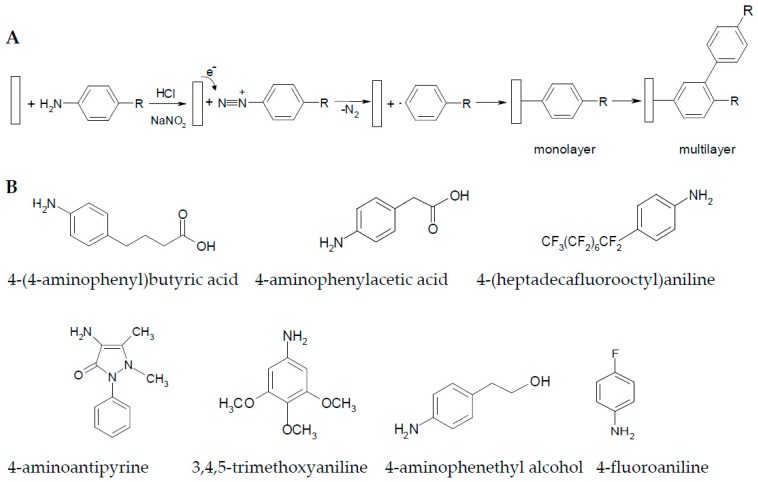
(**A**) Scheme of the electrodeposition reaction used for protective layer formation on copper surface; (**B**) structure of the studied corrosion inhibitors. Amino moieties are removed during the electrodeposition process.

The diazonium organic compounds are usually unstable and can be prepared by a standard diazotisation procedure involving the treatment of aromatic amines with sodium nitrite in the presence of a mineral acid (HCl) [12]. We have electrodeposited organic (multi)layers on the copper surface using the diazonium cations generated in situ from the corresponding amines. One electron transfer from the copper surface to the diazonium salt results radical aryl formation after releasing of N_2_ (Figure 1A). In this study, we have used seven aromatic organic compounds, with various functional groups (such as halogens, acids, alcohol, etc.) or different lengths of alkane chains (Figure 1B), to form organic (multi)layers on copper via diazonium chemistry using their amino moieties. We have chosen such structures because we were interested in the influence of the substitution, as well as in the effect of the electron withdrawing or electron donating subgroups in the phenyl ring.

The corrosion protection properties of the different electrodeposited organic layers on copper surface were investigated, both in acidic or chloride solutions, using potentiodynamic polarization (Tafel curves) and electrochemical noise (ECN) techniques. Furthermore, sodium chloride and citrates are used in some food industrial processes, so it is important to study the influence of these media on the corrosion of copper [13]. The electrodeposited organic films are very useful as coating systems to confer a better grip, stability, and corrosion protection of the topcoat paints [14].

## 2. Results

### 2.1. Influence of the Electrodeposition Process on the Copper Surface

Aryl layers can be strongly attached on copper surface based on the simple electrochemical binding of diazonium salts formed in situ form the corresponding aromatic amines. Various electrodes modified with functionalized surfaces obtained by electrodeposition of diazonium compounds were previously developed and characterized by our group for analytical applications [15,16]. The electrodeposition procedures were adapted from our previous works for optimal inhibition protection of copper surfaces. The reduction of diazonium salts in aqueous solution under constant current conditions ensures a constant electrons flow at the copper surface and can lead to a controlled and reproducible coverage of the substrate [15,16]. The electrode potential varies between −4 and −20 mV (vs. Ag/AgCl) during the electrodeposition of different organic layers. Electrochemical reduction of diazonium salts on electrode surfaces leads to the formation of a covalently grafted mono- or multilayer on the substrate surfaces, depending the modification procedures used, nature of aryldiazonium salt or substrate material (Figure 1A) [17].

A simulation of the surface covering process based on a blank electrodeposition procedure was used to evaluate the eventual effects of electrochemical layer formation process on copper corrosion behavior. The mimicking of the copper surface coating was made using a blank solution prepared with 5 mM NaNO_2_, 50 mM HCl, but in the absence of the studied organic amines. No significant differences were observed between the Tafel polarization curves recorded before and after a simulated electrodeposition for a bare copper surface. It was, thus, demonstrated that the organic layer electrodeposition process does not have significant effects on the copper surface. Thus, the corrosion inhibition observed for covered copper surfaces is due to the protective properties of the organic layers and not attributable to any surface passivation produced by the electrodeposition process.

### 2.2. Electrogravimetric Study of the Layer Formation Process

QCM is a technique useful for monitoring the mass of immobilised molecules via changes in the resonant frequency which can be directly related to the coated organic layer mass on the sensor [18]. The oscillation frequency is decreased by the film coating on the quartz crystal surface. The linear relationship between the deposited mass per unit area and the resonance frequency shift of the QCM is described by the Sauerbrey equation (Equation (1)) [19]:

(1) $$\Delta f=-\frac{2f_{0}^{2}}{A\sqrt{\rho_{q}\mu_{q}}}\Delta m=-C_{f}\Delta m$$

where Δ*f* is the frequency shift, Δ*m* is the mass change, *f*_0_ is the operating frequency of the QCM; A is the active surface area of the QCM electrodes; *ρ*_q_ is the density of the piezoelectric quartz crystal (2.65 g/cm^3^) and μ_q_ is the shear modulus (2.947 × 10^11^ g/cm s^2^). C_f_ is a constant characterizing the sensor crystal (C_f_ = 0.902 Hz/ng for a 9 MHz quartz crystal). The change in frequency of the quartz crystal before, during, and after electrodeposition was measured. Under this condition, the Sauerbrey relationship is used to convert the measured frequency change to a quantified electrodeposed mass.

We have used QCM measurements to estimate the mass of the electrodeposited organic layers. We have observed that the frequency linearly decreases until a saturation is reached. The modification of the measured frequency change from linear to a plateau after at least 300 s, a longer time than the ones used for corrosion inhibition studies. The linear increase of the organic film mass with time demonstrates a continuous layer growth mechanism [20]. The electrodeposition reaction depicted in Figure 1A used for layer formation is dependent on the electrical charge transfer trough copper surface. Under the galvanostatic conditions used in this paper, the electrodeposited mass is dependent on the layer formation time. The calculated masses of the organic layers electrodeposed onto the crystal surfaces for the two different deposition times, 30 s and 300 s, are presented in Table 1. The electrodeposited mass varies between 26 ng/cm^2^ for 4-fluoroaniline to 40 ng/cm^2^ 4-aminoantipyrine in the case of layer formation during 30 s. For the electrodeposition time of 300 s the quantified layer masses varied between 266 ng/cm^2^ for for 4-fluoroaniline to 442 ng/cm^2^ for 4-phenylbutyric acid. The obtained mass of the anticorrosion layers are similar with other inhibitors organic films deposed on aluminum [21] or iron [22] reported in literature. Different organic layers formed by deposition of a significantly higher quantity of modifiers were reported for corrosion inhibition of copper surfaces [23]. 

### 2.3. Electrochemical noise (ECN)

The fluctuations of potential or current occurring at the electrode/solution interface can be investigated using ECN to elucidate the corrosion processes [24,25]. Continuous noise measurements were previously used to study the type of pitting corrosion of the copper covered by organic layers [26]. The pitting index (PI) together with skewness and kurtosis of the time domain data were used to characterize the inhibition corrosion due to organic layers. The pitting index was calculated as shown according to:

(2) $$\mathrm{PI}=\frac{s_{i}}{I_{\mathrm{rms}}}$$

where *σ*_i_ is the standard deviation of the measured current from ECN and I_rms_ is the root mean square value of the current. The pitting index varies between 0 and 1 and indicates uniform corrosion if PI < 0.01, mixed corrosion if 0.01 < PI < 0.1 and localized corrosion for 0.1 < PI < 1 [27]. Skewness and kurtosis of the time domain data are used to identify the type of corrosion [24]. The skewness of a signal represents the deviation degree from the symmetry of a distribution. If it is negative, the curve is shifted to the left, if positive, the curve is shifted for the right. For a symmetrical distribution, the skewness is zero. The kurtosis of the normal distribution is 3. Higher kurtosis values (>3) indicate a sharper distribution of the individual values than the normal distribution (few extreme values form the mean, but with higher deviance), while lower kurtosis values (<3) indicate a flatter distribution (more values different from the mean, but less extreme differences) [28].

**Figure 2:**
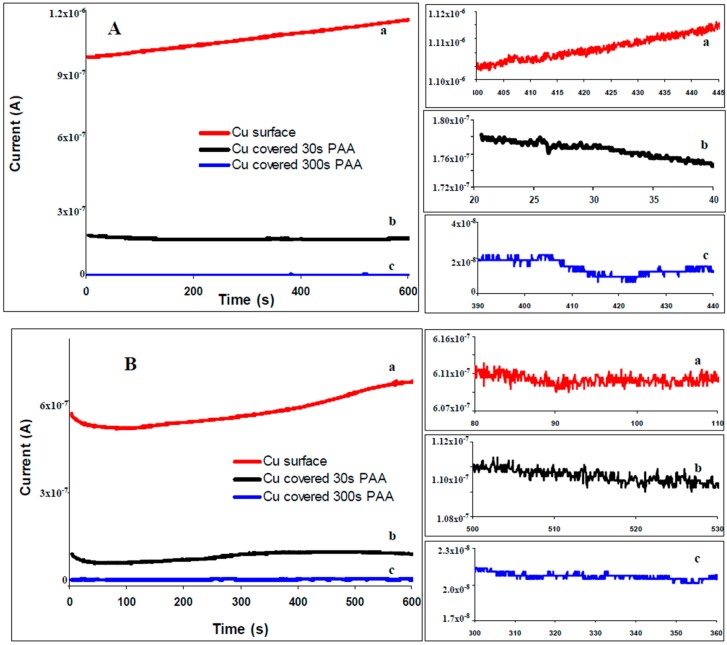
Electrochemical current noise (ECN) recorded for copper: bare (a ─), covered with 4-phenyl acetic acid (PAA) during 30 s (b **─**) and 300 s (c ─). (**A**) ECN signals obtained in buffer citrate solution, pH = 3; (**B**) ECN signals obtained in 3.5% NaCl solution. The inserted images show a zoomed-in region of the of the recorded ECN signals.

Typical ECN time records of the current fluctuation recorded for bare copper electrodes and electrodes covered with 4-phenyl acetic are depicted in Figure 2A for 0.1 M citric buffer solution and in Figure 2B for 3.5% NaCl. Current noise time series for bare copper surfaces are characterized by higher values than current noise recorded for copper-4-phenyl acetic electrode which is a qualitative indication of effectiveness of the inhibitor. The zooming of the signals is characteristic for the corrosion pits. 

The ECN transients had preponderantly shapes characteristic for type II transients [29]. The recorded signals for the bare copper surfaces have higher amplitude fluctuations (Figure 2A-a) in comparison with modified surfaces. The magnitude of the signals recorded for modified surfaces decreases with deposition time (higher Figure 2A-b for 30 s than Figure 2A-c for 300 s). The signals represented in Figure 2 are representative for all of the organic compounds (see Figures S1 and S2). It was observed that the magnitude of all ECN signals recorded for copper surfaces covered with any of the seven tested compounds decreases with the increase of deposition time and is greatly smaller in comparison with bare copper surfaces.

**Figure 3:**
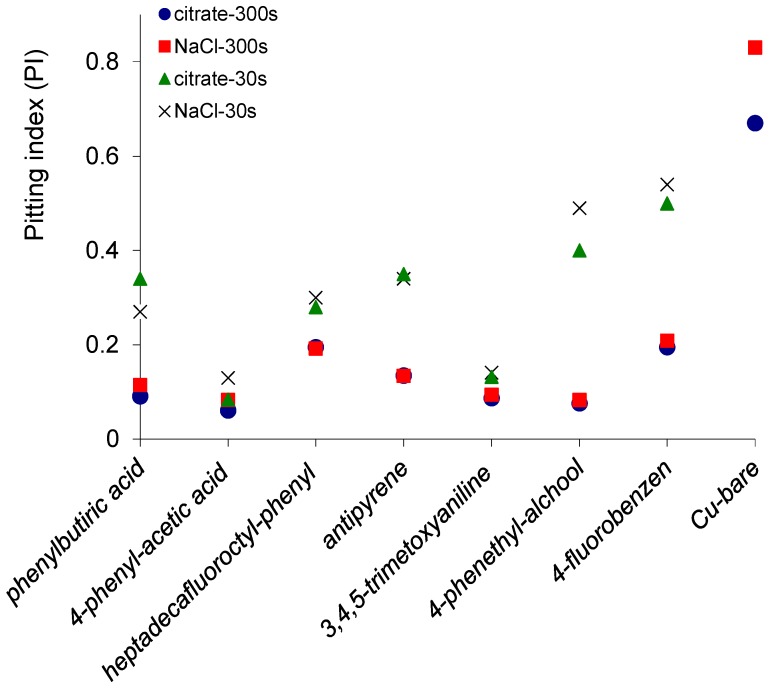
The pitting index obtained for copper surfaces covered with organic layers electrodeposited for 30 and 300 s in citrate buffer and saline solution. For comparison is presented also the pitting index measured for bare copper.

The relationship between the potential and current fluctuations has been evaluated. The pitting index (PI), skewness and kurtosis of potential and current noises for copper exposed to 3.5% NaCl or citrate buffer pH = 3.00 determined by ECN are presented in Table S1 and Figure 3. The values of statistical parameters in the Table S1 indicate a mixed or pitting (localized) corrosion mechanism [27,30]. For the bare copper surfaces, it was observed a marked localized (pitting) corrosion with PI = 0.67 for citrate buffer pH = 3.00 and PI = 0.83 for 3.5% NaCl solution. The pitting indices indicate a predominantly localized corrosion for 30 s electrodeposition time for copper surfaces covered with all seven organic inhibitors [27], but the values of PI were substantially smaller. A mixed corrosion was observed for the thicker layers of phenylacetic acid, phenethyl alcohol, phenylbutyric acid and trimethoxyphenyl obtained by 300 s electrodeposition. Thus, it was observed that the electrodeposition of the organic layer had a general effect of the reducing of the PI and transformation of the pitting corrosion of the bare copper surface to a mixt type corrosion. The kurtosis values for both current and potential are higher for the copper surfaces modified using 4-(heptadecafluorooctyl)aniline, 4-fluoroaniline and 4-aminophenethyl alcohol. The kurtosis values are also higher mainly in saline solution than citrate buffer for the same surface, corresponding to spikier distributions that are associated with a pitting corrosion process.

### 2.4. Potentiodynamic Polarization Measurements 

**Figure 4:**
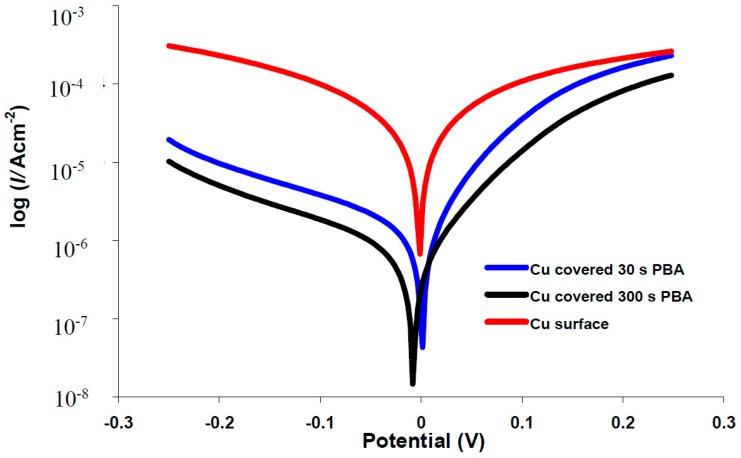
Typical polarization curves for corrosion in citrate buffer, pH3 of copper electrode (─), covered with 4-phenylbutyric acid (PBA): 30 s (─) and 300 s (─).

**Figure 5:**
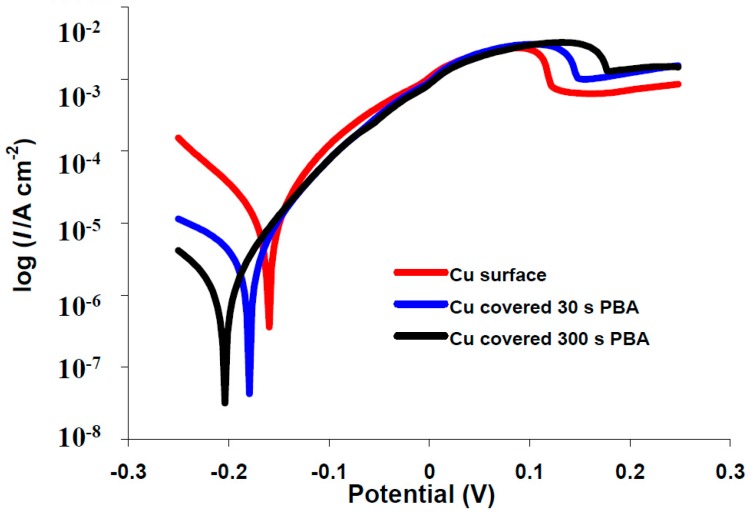
Typical polarization curves for corrosion in 3.5% NaCl of copper electrode (─), covered with 4-phenylbutyric acid (PBA): 30 s (─) and 300 s (─).

The effects of the electrodeposited organic layers on the kinetics of the anodic/cathodic reactions and corrosion inhibitors efficiencies were studied with potentiodynamic polarization measurements. The polarization curves for bare copper and covered with phenylacetic acid in buffer citrate (pH = 3.00) or 3.5% NaCl solutions are represented in Figure 4 and Figure 5. Tafel plots recorded for all of the other investigated organic compounds are similar with phenylacetic acid (see Figures S3 and S4). It is obvious from the figure that corrosion current density decreases for modified surfaces and that the blocking effect of organic layer is bigger for a 300 s electrodeposition in comparison with 30 s. Two different areas are distinguished in the anodic polarization curves recorded in 3.5% NaCl: up to ~0.1 V is an active process which electrode behavior shows an electrochemical active dissolution of the copper. The second area of the anodic Tafel curve is a passivation with a current limitation (Figure 4 and Figure S4).

The electrochemical parameters such as corrosion potential (*E*_corr_), corrosion current density (*i*_corr_), cathodic Tafel slope (*β*_c_), anodic Tafel slope (*β*_a_), polarization resistance (*R*_p_), and inhibition efficiency (*IE*) were calculated from Tafel plots and are presented in Table S2. The *IE* was calculated from polarization measurements according to the relation given below [31]:

(3) $$IE(\%)=\frac{i_{\mathrm{corr}}-i_{\mathrm{corr}}^{\mathrm{inh}}}{i_{\mathrm{corr}}}\times 100$$

were *i*_corr_ and $i_{\mathrm{corr}}^{\mathrm{inh}}$ are uninhibited and inhibited corrosion current densities.

**Figure 6:**
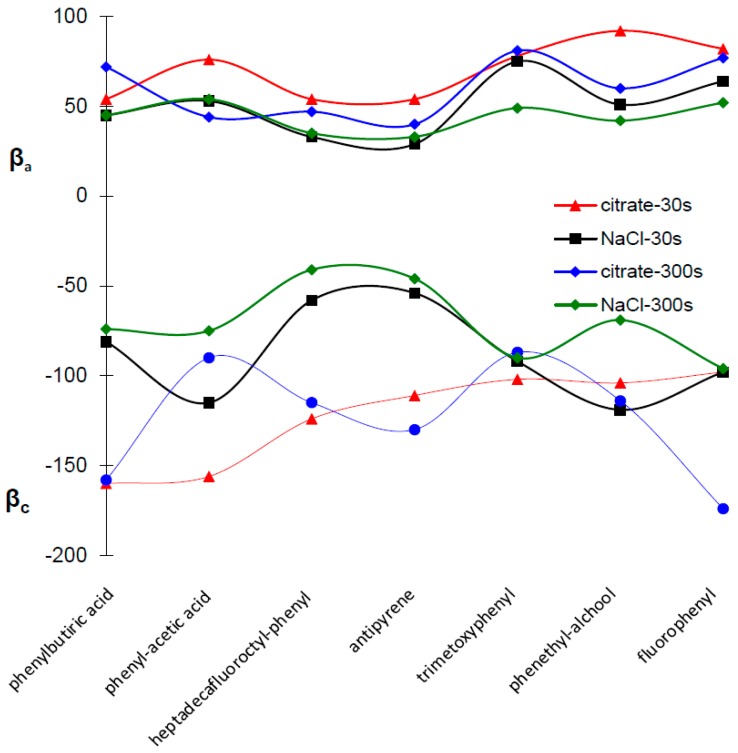
Cathodic Tafel slope (*β*_c_) and anodic Tafel slope (*β*_a_) for the copper surfaces covered with electrodeposited organic layers.

The *E*_corr_ values measured in 3.5% NaCl are shifted with approximately 175 mV towards more negative potentials in comparisons with experiments carried out in buffer citrate pH = 3.00 for all investigated electrodes. The organic layer is considered to be a cathodic or anodic type inhibitor (corresponding to a potential shifts towards the negative or positive direction, respectively) if the inhibitor induces a modification of the *E*_corr_ greater than 85 mV in comparison with the bare surface [32]. If the displacement in *E*_corr_ is smaller than 85 mV, then the inhibitor mechanism can be seen as a mixed type [32]. The displacement of the *E*_corr_ measured for copper surfaces covered by an organic layer is a small shift compared to bare surfaces (between +8 and −46 mV in 3.5% NaCl and between +4 and −31 mV for citrate buffer, pH = 3.00), which is an indication that all the studied organic inhibitors act as a mixed-type inhibitor. The comparison of Tafel slopes for bare and covered electrodes reveals that, for all corrosion inhibitors, both the cathodic (*β*_c_) and anodic slopes (*β*_a_) were reduced with a variable magnitude that did not show any definite trend in function of the layer depostion time or corrosion media (Figure 6). The modification of Tafel slopes suggests also a mixed-type control of the corrosion. The corrosion mechanism of copper is well known and it is based on a complex anodic dissolution and oxygen reduction [33]. The aggressive chloride ions promote cathodic corrosion and the formation of organic layers on copper surfaces are reported to be an efficient barrier for both to copper dissolution and O_2_ reduction [33].

The polarization resistance (*R*_p_) is the transition resistance between the electrodes and the electrolyte and was determined from Tafel plots according to Stern-Geary equation [34]:

(4) $$R_{p}=\frac{\beta_{a}\beta_{c}}{2.303(\beta_{a}+\beta_{c})}\times \frac{1}{i_{\mathrm{corr}}}$$

The values *R*_p_ increase for all the modified copper surfaces in comparison with bare copper electrode and also for 300 s electrodeposition are higher in comparison with 30 s. This indicates the anti-corrosion efficacy of the electrodeposed layers and the fact that a longer deposition time is more effective in corrosion inhibition. 

**Figure 7:**
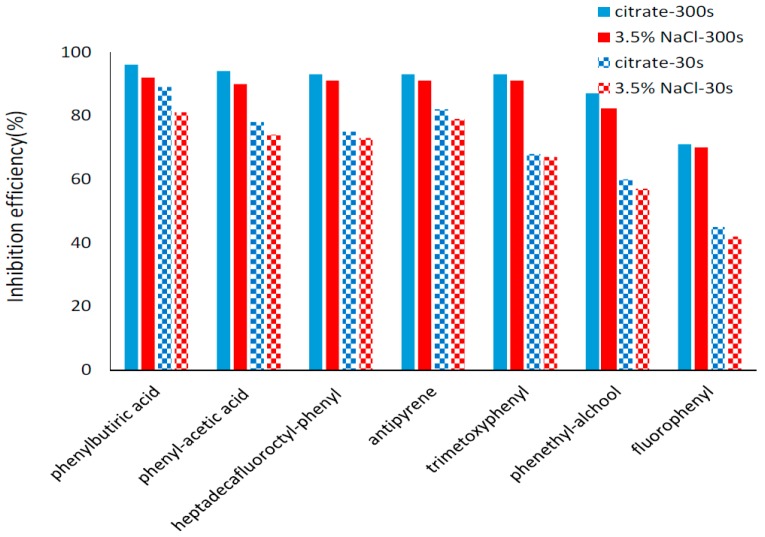
Inhibition efficiencies for the investigated electrodeposited organic layers.

The *i*_corr_ were determined by extrapolation of the linear part for the cathodic and anodic Tafel plots and the corrosion rates were calculated in relation to electrode area. It can be seen from Table S2 that *i*_corr_ obtained for the copper modified surfaces is decreasing for longer electrodeposition time for all investigated organic layers. The *i*_corr_ values obtained for bare copper surfaces are significantly higher than the ones measured for modified surfaces. Correlated with the variation of the *i*_corr_, the inhibition efficiency was found to increase with increasing electrodeposition time for all the studied inhibitors due to the formation of thicker protective layers. Remarkable inhibition efficiencies (higher than 90% in both corrosion media) were found for five of the tested organic compounds using 300 s deposition time: 4-aminophenyl acetic acid, 4-(heptadecafluorooctyl)aniline, 4-(4-aminophenyl)butyric acid, 4-aminoantipyrine and 3,4,5-trimethoxyaniline. The best performing organic compound in both corrosion media was 4-(4-aminophenyl)butyric acid while two compounds have inhibition efficiencies less than 90%: 4-aminophenethyl alcohol and 4-fluoroaniline. The inhibition efficiencies were slightly higher in citrate buffer, pH = 3.00 in comparison with 3.5% NaCl for all tested compounds (Figure 7). Thus, for the top five performing compounds deposed during 300 s, the corrosion inhibition efficiencies varies between 93% and 96% in citrate buffer, pH = 3.00 while in 3.5% NaCl the inhibition is 90%–92%. The inhibitive action of organic coating on copper surface improve its corrosion resistance and the efficiency reaches over 90%. The layers formed by self-assembled monolayers (SAMs) on the copper surface achieved a good protection, but the process requires a long immersion time: 1 [9] 12 [35] or 24 h [36]. Thus, diazonium surface coverage is a rapid and available anti-corrosion method. 

It was observed that the substituent groups play an important role in determining the extent of corrosion protection among the different inhibitors. The substituents influence the inhibition corrosion of copper by inducing various electronic effects that influence both the electric properties of the organic layer and the interactions of the organic compounds with copper surface [9]. The highest inhibition efficiencies in citrate buffer, pH = 3.00 was obtained for the two compounds that have carboxyl groups: 4-aminophenyl acetic acid and 4-(4-aminophenyl)butyric acid. The enhancement of protection efficiency of the chosen carboxylic acids inhibitor layers may be attributed to the inductive effect of the functional groups [37]. It can be observed that the inhibition efficiency slightly increases with longer chain of the carboxylic acid radical. Additinoally, the molecule with –OH substituent from phenetyl alcohol show weaker anticorrosion ability in comparison with –COOH. One difference between the two functional groups is that the inductive effect of -OH decreases in magnitude than the –COOH. 

Substituents attached to an aromatic ring influences the π electron density in the phenyl ring. Due to the inductive effect a methoxy groups (–OCH_3_) from trimethoxyphenyl layer the electron density on the phenyl ring, thereby increasing their inhibitive property. On the other hand, the presence of these groups both in mega or para position have strong resonance effect.

The lowest inhibiting effect had p-fluoroaniline (~70%) and this can be attributed to the presence of electron-withdrawing fluorine atom in compound [37]. On the other hand, the 4-(heptadecafluorooctyl)aniline has significantly better anticorrosion efficacy than p-fluoroaniline due to differences in structure both in number of substituents and length of the side chain. Moreover, this compound is used for preparation of fluorinated polymers [38] which can be very effective as a coating systems [14].

## 3. Materials and Methods 

### 3.1. Experimental Materials

4-aminophenylacetic acid, 4-aminophenethyl alcohol, 4-fluoroaniline, 4-(heptadecafluorooctyl)aniline, 4-aminoantipyrine, 4-(4-aminophenyl)butyric acid, and 3,4,5-trimethoxyaniline, sodium nitrite, hydrogen peroxide 30%, sodium chloride, sodium citrate, and citric acid were obtained from Sigma-Aldrich (Taufkirchen, Germany). CuSO_4_ anhydrous 98% was purchased from Alfa Aesar (Karlsruhe, Germany). Hydrochloric acid (37%) was obtained from Carl Roth (Karlsruhe, Germany). Sulphuric acid (98%) was purchased from Merck Millipore (Darmstadt, Germany). Chemically pure acetonitrile, ethanol, and purified water (18 MΩ cm^−1^, Millipore Darmstadt, Germany) were used to prepare solutions.

### 3.2. Apparatus

All electrochemical measurements (chronoamperometry, chronopotentiometry, and linear sweep voltammetry) were carried out using a PGSTAT302N potentiostat/galvanostat (Metrohm-Autolab, Utrecht, The Netherlands) equipped with a conventional three-electrode cell and controlled using Nova 1.8 software. The working electrode was a gold screen-printed electrode (SPE) 4 mm in diameter from DropSens (C2XXAT, Llanera, Spain), the reference electrode was Ag/AgCl//3M KCl (Metrohm-Autolab, 210 mV vs. standard hydrogen electrode) and platinum foil was used as an auxiliary electrode. The linear polarization curves were recorded at the sweep rate of 1 mV/s in the scanning range of −250 mV to +250 mV vs. Ag/AgCl. Electrochemical noise (ECN) measurements were recorded during 600 s using an Autolab ECN module in galvanostatic mode with automatic current range up to 100 μA. Both current and voltage noise were measured simultaneously on an electrochemical cell under control of a zero resistance ammeter. Two identical electrodes were used for data acquisition and a silver chloride electrode for reference. The cell was placed into a Faraday cage for both measurements of polarization curves and ECN. All corrosion measurements were carried out in presence of oxygen, at room temperature, using two solutions: (i) 3.5% NaCl with pH = 4.77 and (ii) 0.1 M citrate buffer pH = 3.00.

A quartz crystal microbalance (QCM) measurements of the organic layer electrodeposition process were carried out using a QCM 922 (Princeton Applied Research) controlled by WinEchem software. AT-cut quartz crystals with 9 MHz frequency coated with mirror polished gold electrodes on both sides (surface area of 0.2 cm^2^, thickness gold layer of approximately 300 nm) were purchased from Ametek (Berwyn, PA, USA).

An Autolab pX1000 module was used for pH measurements.

### 3.3. Copper Electrodeposition on Gold SPE

Before electrochemical modification, the gold screen printed electrodes were electrochemically cleaned by cycling between −0.6 and +0.6 V, at 100 mV/s in 0.5 M H_2_SO_4_ solution. The electrochemical deposition of copper by chronoamperometry were performed at 1 mA for 100 s in 0.5 M CuSO_4_ and 0.5 M H_2_SO_4_ using a copper wire as anode and the gold screen printed electrode as cathode.

### 3.4. Electrodeposition of Aryl Diazonium Salts on Copper Surfaces

The procedure for modifying the copper surface is based on the reduction of diazonium salt. Before derivatization, the electrodes were rinsed with water and dried under a stream of argon. In situ generation of diazonium salts from amino groups was made under ice immediately before electrodeposition in the presence of 5 mM NaNO_2_, 50 mM HCl, and 5 mM amino-containing organic compounds (one of the seven studied). Electrochemically reductive adsorption of aryl diazonium salts on electrode surface is based on a classical radical mechanism and leads to covalent modification. The electrochemical deposition of various diazonium compounds was made by chronopotentiometry at −0.25 mA for 30 or 300 s. 

### 3.5. Electrodeposition of Aryl Diazonium Salts on Copper Surfaces

The piezoelectric quartz crystal was cleaned with a solution of 1:1 H_2_SO_4_, 98%: H_2_O_2_, 30% *v*/*v*, for 15 min, rinsed with distilled water and dried with an argon stream. The crystal was mounted into the cell with one side in contact with the solution and connected to the potentiostat as a working electrode of the electrochemical cell, while the other side was in air. The stability of the frequency was verified before and after the electrodeposition process to avoid false signals due to any signal drift produced by external factors such as temperature [39]. The electrogravimetric monitoring of the organic formation was performed using the electrodeposition conditions described in Section 2.4. Deposition time by piezoelectric analysis method was 600 s.

## 4. Conclusions

The corrosion protection action of organic layers electrodeposited on a copper surface using electroreduction of diazonium salts was demonstrated for seven organic compounds. The ECN was used to study the type of corrosion. The linear polarization measurements provided a convenient way to investigate inhibitors efficiencies of the organic layers. Notably, layers with good corrosion inhibition efficiency were obtained with an electrodeposition time of only 300 s. Remarkably, the achieved inhibition efficiencies exceeded over 90% for five of the seven tested organic compounds, 4-(4-aminophenyl)butyric acid being the top performer in both 3.5% NaCl and citrate buffer, pH = 3.00. As well, a dependence of the inhibition efficiency with the chemical nature of the substituents present in the aromatic compounds was found. Interestingly, the electrodeposited layers have a good adhesion due to chemical bonds and could be further used as a primer in a multiple-layer protective strategy.

## Figures and Tables

**Table 1:** The mass of the electrodeposited organic layers measured using QCM.

Organic Compound	Δ*m*/cm^2^ (ng/cm^2^)	
	Electrodeposition Time 30 (s)	Electrodeposition Time 300 (s)
4-aminophenylacetic acid	39	388
4-aminophenethyl alcohol	29	274
4-fluoroaniline	26	266
4-(heptadecafluorooctyl)aniline	36	377
4-aminoantipyrine	40	416
4-phenylbutyric acid	34	442
3,4,5-trimethoxyaniline	38	435

## Supplementary Materials

The following are available online at www.mdpi.com/1996-1944/10/3/235/s1.
