# Supplementary material for: Electrodeposited Organic Layers Formed from Aryl Diazonium Salts for Inhibition of Copper Corrosion
Source: Materials (Basel). 2017 Feb 28;10(3):235. doi: 10.3390/ma10030235 (PMC5503370; doi:10.3390/ma10030235)
Supplement: Supplementary file 1 [file materials-10-00235-s001.pdf]

Electronic Supplementary Material

Article

Copper corrosion inhibition using electrodeposited organic layers formed from aryl diazonium salts

Ana Chira [1], Bogdan Bucur [1*] and Gabriel-Lucian Radu [1,]

[1] National Institute of Research and Development for Biological Sciences, Centre of Bioanalysis, 296 Splaiul Independentei, 060031 Bucharest, Romania

* Correspondence: bucurica@yahoo.com; Tel.: +40-021-22-00-900;

Table S1. Electrochemical noise results for copper surfaces modified with organic layers.

Surface modification layer (electrodeposition time)	Corrosive media	Skewness		Kurtosis		Pitting index
		E	I	E	I	
Cu-4-phenyl-acetic acid (30 s)	citrate buffer, pH=3	-1.06	1.99	2.75	1.54	0.084
	3.5% NaCl	-0.82	-0.38	2.15	1.46	0.13
Cu-4-phenyl-acetic acid (300 s)	citrate buffer, pH=3	-0.49	-0.9	1.97	2.60	0.061
	3.5% NaCl	1.77	-1.17	2.70	2.86	0.083
Cu-4-phenethyl-alcohol (30 s)	citrate buffer, pH=3	-1.59	-2.39	3.28	2.99	0.40
	3.5% NaCl	-1.61	-0.22	4.86	1.68	0.49
Cu-4-phenethyl-alcohol (300 s)	citrate buffer, pH=3	0.71	0.68	2.28	2.15	0.076
	3.5% NaCl	-0.58	0.35	2.15	1.64	0.083
Cu-4-fluorophenyl (30 s)	citrate buffer, pH=3	-2.43	0.43	3.43	2.01	0.50
	3.5% NaCl	-2.74	1.10	4.1	2.61	0.57
4-fluorophenyl (300 s)	citrate buffer, pH=3	-1.41	0.62	3.21	3.75	0.195
	3.5% NaCl	-1.47	-1.34	4.95	4.37	0.209
Cu-4-heptadecafluoroctyl-phenyl (30 s)	citrate buffer, pH=3	-2.86	-1.57	2.9	4.74	0.283
	3.5% NaCl	-2.45	1.51	4.7	5.04	0.30
Cu-4-heptadecafluoroctyl-phenyl (300 s)	citrate buffer, pH=3	-2.54	-2.93	3.90	4.64	0.195
	3.5% NaCl	-2.08	-2.11	4.35	4.81	0.192
Cu-antipyrene (30 s)	citrate buffer, pH=3	0.68	0.49	2.13	3.31	0.35
	3.5% NaCl	0.67	0.27	2.95	2.39	0.34
Cu-antipyrene (300 s)	citrate buffer, pH=3	0.15	0.05	2.73	1.57	0.135
	3.5% NaCl	-0.28	-1.46	1.95	3.05	0.134
Cu-4-phenylbutiric acid (30 s)	citrate buffer, pH=3	0.81	1.92	2.22	2.44	0.34
	3.5% NaCl	-0.68	-0.72	2.17	2.49	0.27
Cu-4-phenylbutiric acid (300 s)	citrate buffer, pH=3	-0.79	-0.91	2.28	2.37	0.091
	3.5% NaCl	-0.54	0.21	1.72	1.37	0.115
Cu-3,4,5-trimetoxyphenyl (30s)	citrate buffer, pH=3	-2.14	2.59	3.87	3.63	0.132
	3.5% NaCl	-0.77	-0.15	3.05	1.61	0.141
Cu-3,4,5-trimetoxyphenyl (300 s)	citrate buffer, pH=3	0.05	-1.31	1.61	4.57	0.087
	3.5% NaCl	0.62	-1.06	2.01	2.97	0.094
Cu (bare electrode for comparison)	citrate buffer, pH=3	0.25	0.28	1.90	1.79	0.67
	3.5% NaCl	0.71	0.68	2.28	2.15	0.83

Table S-2. Potentiodynamic polarization parameters and inhibition efficiencies obtained for copper electrodes covered with protective layers.

Surface modification layer (electrodeposition time)	Corrosive media	Slope β_c mV/dec	Slope β_a mV/dec	E_{corr} (mV)	Polarization resistance [kΩ/cm²]	Corrosion rate [mm/year]	I_{corr} [µA/cm²]	*IE* (%)
Cu-4-phenyl-acetic acid (30 s)	citrate buffer, pH=3	-156	76	-16	86.9	1.20	52.2	78
	3.5% NaCl	-115	53	-178	72.7	1.03	44.8	74
Cu-4-phenyl-acetic acid (300 s)	citrate buffer, pH=3	-90	44	-32	158.3	0.38	16.6	94
	3.5% NaCl	-75	54	-203	169.4	0.38	16.5	90
Cu- 4-phenethyl-alcohol (30 s)	citrate buffer, pH=3	-104	92	-3	36.3	2.79	118.3	60
	3.5% NaCl	-119	51	-178	70.7	1.03	44.5	57
Cu- 4-phenethyl-alcohol (300 s)	citrate buffer, pH=3	-114	60	-4	159.3	0.84	38.4	87
	3.5% NaCl	-69	42	-206	136.1	0.39	17.1	82
Cu-4-fluorophenyl (30 s)	citrate buffer, pH=3	-98	82	-10	29.4	3.10	134.8	45
	3.5% NaCl	-98	64	-150	14.7	2.96	129.1	42
Cu-4-fluorophenyl (300 s)	citrate buffer, pH=3	-174	77	-16	44.4	1.61	70.2	71
	3.5% NaCl	-96	52	-161	46.1	1.54	66.9	70
Cu-heptadecafluoroctyl-phenyl (30 s)	citrate buffer, pH=3	-124	54	-20	59.6	1.29	56.3	75
	3.5% NaCl	-58	33	-180	37.7	1.13	77.2	73
Cu- heptadecafluoroctyl-phenyl (300 s)	citrate buffer, pH=3	-115	47	-21	201.9	0.34	14.8	93
	3.5% NaCl	-41	35	-205	108.7	0.35	16.5	91
Cu-antipyrene (30 s)	citrate buffer, pH=3	-111	54	-20	72.9	1.03	44.8	82
	3.5% NaCl	-54	29	-200	72.9	0.53	23.1	79
Cu-antipyrene (300 s)	citrate buffer, pH=3	-130	40	-21	150.0	0.41	18.0	93
	3.5% NaCl	-46	33	-223	151.4	0.24	10.4	91
Cu-phenylbutiric acid (30 s)	citrate buffer, pH=3	-160	54	1	144.3	0.33	23.4	89
	3.5% NaCl	-81	45	-179	176.9	0.77	33.5	81
Cu-phenylbutiric acid (300s)	citrate buffer, pH=3	-158	72	-6	180.0	0.17	7.6	96
	3.5% NaCl	-74	45	-203	172.9	0.33	14.4	92
Cu-trimetoxyphenyl (30s)	citrate buffer, pH=3	-102	78	3	27.7	1.82	79.3	68
	3.5% NaCl	-92	75	-190	47.6	1.18	51.5	67
Cu-trimetoxyphenyl (300 s)	citrate buffer, pH=3	-87	81	-13	170.0	0.40	17.7	93
	3.5% NaCl	-90	49	-195	140.9	0.33	14.4	91
Cu (bare electrode for comparison)	citrate buffer, pH=3	-147	126	-1	20.7	4.89	213.7	-
	3.5% NaCl	-82	61	-160	17.6	4.06	176.3	-

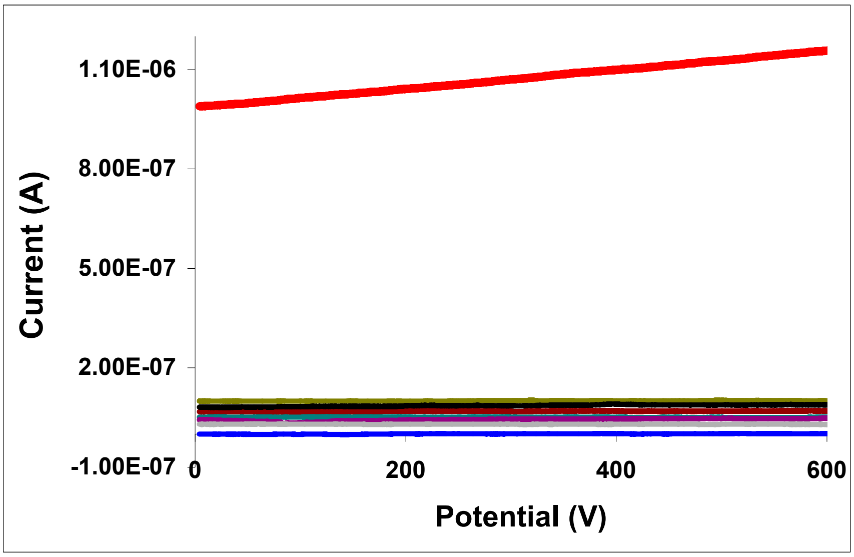

Figure S1. Electrochemical current noise recorded in buffer citrate solution, pH=3 for copper: bare (—) or covered during 300s with 4-phenylbutyric acid (—), antipyrine (—), 4-(heptadecafluorooctyl)benzen (—), 3,4,5-trimethoxybenzen (—),4-phenyl acetic acid (—), 4-phenethyl alchool (—), 4-fluorobenzen (—).

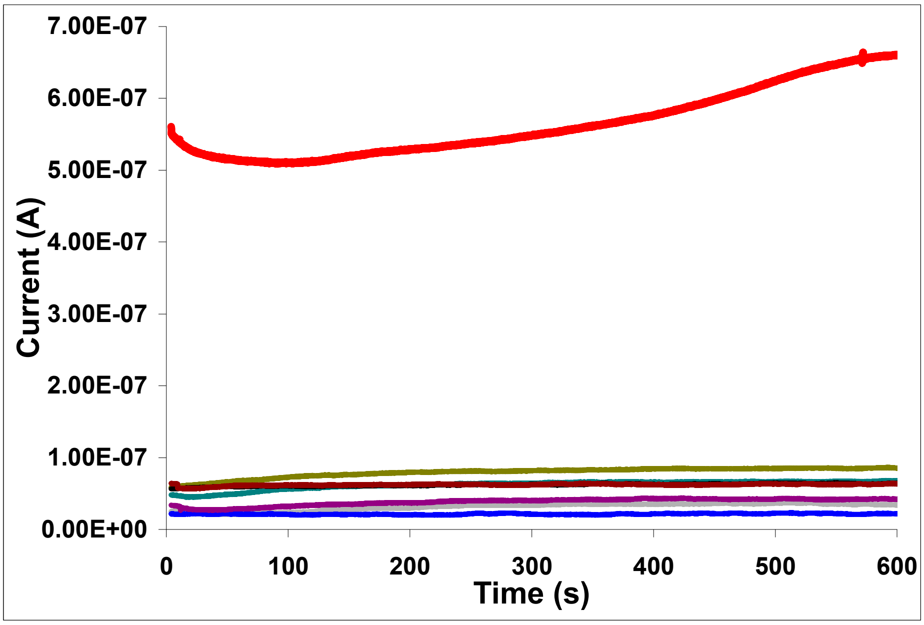

Figure S2. Electrochemical current noise recorded in buffer in 3% NaCl for copper: bare (—) or covered during 300s with 4-phenylbutyric acid (—), antipyrine (—), 4-(heptadecafluorooctyl)benzen (—), 3,4,5-trimethoxybenzen (—),4-phenyl acetic acid (—), 4-phenethyl alchool (—), 4-fluorobenzen (—).

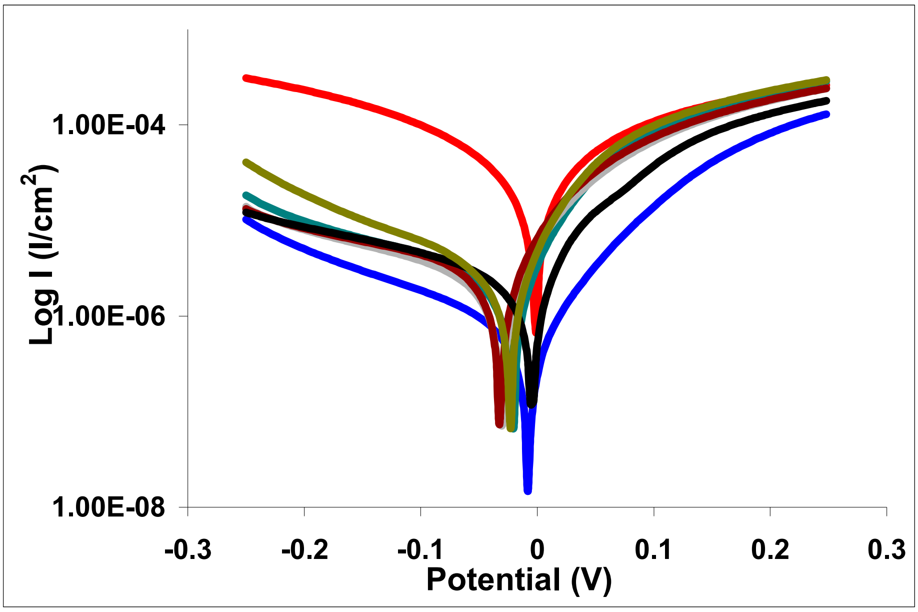

Figure S3. Typical polarization curves for corrosion in buffer citrate of copper: bare (—) or covered during 300s with 4-phenylbutyric acid (—), antipyrine (—), 4-(heptadecafluorooctyl)benzen (—), 3,4,5-trimethoxybenzen (—),4-phenyl acetic acid (—), 4-phenethyl alchool (—), 4-fluorobenzen (—).

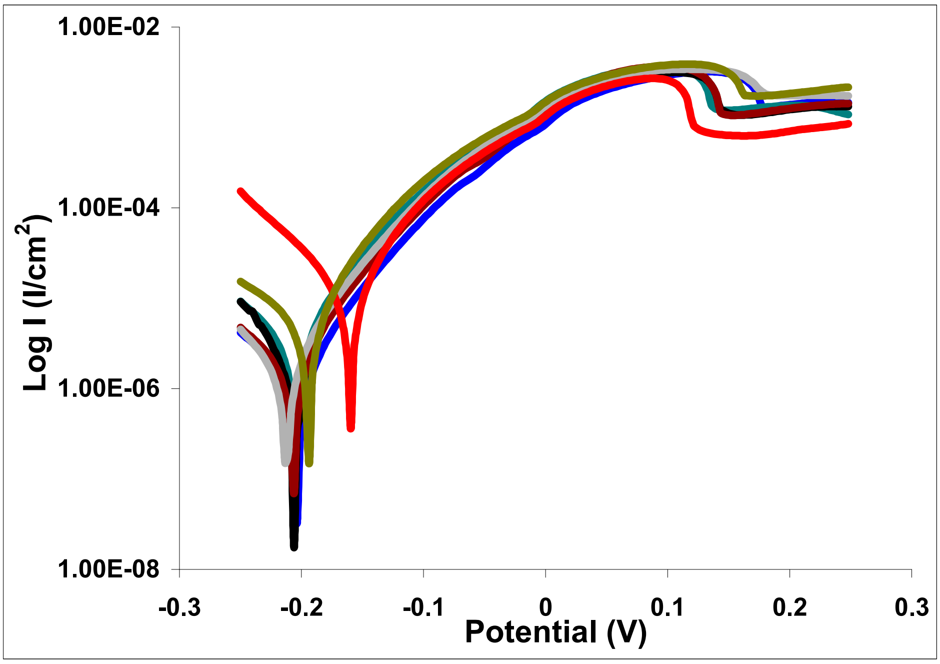

Figure S4. Typical polarization curves for corrosion in 3.5% NaCl solution of copper: bare (—) or covered during 300s with 4-phenylbutyric acid (—), antipyrine (—), 4-(heptadecafluorooctyl)benzen (—), 3,4,5-trimethoxybenzen (—),4-phenyl acetic acid (—), 4-phenethyl alchool (—), 4-fluorobenzen (—).
